# Caseworker Cultural Mediator Involvement in Neurocritical Care for Patients and Families With Non-English Language Preference: A Quality Improvement Project

**DOI:** 10.7759/cureus.37687

**Published:** 2023-04-17

**Authors:** Abhijit V Lele, Anna Brooks, Lea Ann Miyagawa, Asmeret Tesfalem, Kim Lundgren, Rosemary E Cano, Niuvus Ferro-Gonzalez, Yodit Wongelemegist, Anab Abdullahi, John T Christianson, Jeniffer S Huong, Piper L Nash, Wei-Yun Wang, Christine T Fong, Marie-Angele Theard, Sarah Wahlster, Gemi E Jannotta, Monica S Vavilala

**Affiliations:** 1 Anesthesiology and Pain Medicine, Harborview Injury Prevention and Research Center, Harborview Medical Center, University of Washington, Seattle, USA; 2 Neurological Surgery, Harborview Injury Prevention and Research Center, Harborview Medical Center, University of Washington, Seattle, USA; 3 Medicine, Harborview Injury Prevention and Research Center, Harborview Medical Center, University of Washington, Seattle, USA; 4 Interpreter Services, Harborview Medical Center, Seattle, USA; 5 Anesthesiology and Pain Medicine, Harborview Medical Center, University of Washington, Seattle, USA; 6 Neurology, Harborview Medical Center, University of Washington, Seattle, USA; 7 Neurological Surgery, Harborview Medical Center, University of Washington, Seattle, USA; 8 Neurocritical Care Service, Harborview Medical Center, University of Washington, Seattle, USA

**Keywords:** culture and sensitivity, quality improvement research, neurocritical care, implementation, mediators, interpreters

## Abstract

Objective

To describe Harborview Medical Center's experience with the involvement of caseworker cultural mediators (CCM) for patients requiring neurocritical care.

Methods

Using univariate and multivariate analysis (model adjusted for age, Glasgow Coma Scale score (GCS), Sequential Organ Failure Assessment (SOFA) Scores, mechanical ventilation, transition to comfort measures only (CMO), and death by neurologic criteria), we examined CCM team members' involvement in the care of Amharic/Cambodian/Khmer/Somali/Spanish/Vietnamese patients admitted to our neurocritical care service between 2014-2022, factors associated with CCM utilization, and changes in CCM utilization after a QI initiative was implemented in 2020 to encourage healthcare providers to consult the CCM team.

Results

Compared to eligible patients (n=827) who did not receive CCM referral, patients with CCM involvement (n=121) were younger (49 [interquartile range, IQR 38,63] vs. 56 [IQR 42,68] years, p = 0.002), had greater illness severity (admission GCS 8.5 [IQR 3,14] vs. 14 [IQR 7,15], p < 0.001, SOFA scores (5 [IQR 2,8] vs. 4 [IQR2,6], p = 0.007), and more frequently required mechanical ventilation (67% vs. 40%, odds ratio, OR 3.07, 95% CI 2.06,4.64), with higher all-cause mortality (20% vs. 12%, RR 1.83, 95% CI 1.09, 2.95), and with a higher rate of transition to CMO (11.6% vs. 6.2%, OR 2.00, 95% CI 1.03;3.66). The CCM QI initiative was independently associated with increased CCM involvement (aOR 4.22, 95% CI [2.32;7.66]). Overall, 4/10 attempts made by CCMs to reach out to the family to provide support were declined by the family. CCMs reported providing cultural/emotional support (n=96, 79%), end-of-life counseling (n=16, 13%), conflict mediation (n=15, 12.4%), and facilitating goals of care meetings (n=4, 3.3%).

Conclusions

Among eligible patients, CCM consultations appeared to occur in patients with higher disease severity. Our QI initiative increased CCM involvement.

## Introduction

Patient-centered care (PCC) is essential to improving patient experiences and care quality, as it considers individual patient preferences, needs, and values. Cultural differences and language barriers in acutely hospitalized patients can result in multiple challenges in patient care and communication, rendering patients and their families more vulnerable at a critical time. Medical interpretation alone may not always sufficiently address the complex needs of members of immigrant communities [1]. Increasing evidence suggests that our effectiveness as physicians is related to our ability to understand and connect with our patients, including their language and cultural and religious aspects [2].

At our institution, culturally competent care of our increasingly diverse patient population, responsiveness to patients’ value-based needs, and a patient-centered approach to care are crucial aspects of high-quality care. The primary mission of Harborview Medical Center is to provide healthcare for the most vulnerable residents of King County, including immigrant communities and populations with Non-English language preference (NELP) [3]. Our institution utilizes a Caseworker Cultural Mediator (CCM) program to bridge care and communication gaps for these populations [4]. This CCM service serves NELP populations in five languages (Amharic, Cambodian/Khmer, Somali, Spanish, and Vietnamese: i.e., CCM consultation eligible patients).

In addition to medical interpretation, CCM provides patient, healthcare provider, and community support. CCM contributes to the well-being and support of immigrant and refugee patients, families, and communities through a partnership that promotes culturally sensitive care by 1) providing interpretation services, 2) forming and maintaining partnerships with patients and their families, and facilitating their interactions with hospital staff, 3) providing information about cultural norms [5] and perceptions around health, illness, and community resources to healthcare providers, 4) advocating for patients and their families and their needs in the healthcare setting, and 5) community outreach to provide healthcare-related education. CCM services have important goals: a) supporting patients and their families during their hospitalization, b) facilitating therapeutic relationships and trust between patients, families, and the healthcare team [6]. Their involvement may benefit patients and the healthcare system and may be helpful to hospitals that care for socially and culturally diverse populations [7]. While there is data on the successes of cultural case workers in community outreach programs, specifically in increasing prenatal visits and reduction in HbAIc levels due to educational visits by bicultural/bilingual community health workers [8], there are no studies examining the involvement of CCMs in neurocritical care (NCC).

When faced with acute neurological emergencies and life-or-death decisions facing prognostic uncertainty, it is critical to consider cultural norms and perceptions while communicating with patients and families [9]. Based on two index cases involving NELP patients’ families and surrogate decision-makers experiencing conflict, leading to emotional distress for both the families and neurocritical care providers, a 3-phase quality improvement (QI) project was implemented in 2020 to 1) identify deficiencies and inconsistencies in end-of-life care and then to 2) develop and implement a consensus-based initiative to incorporate CCM involvement on our neurocritical care service. This article describes our experience developing and implementing the initiative to provide culturally sensitive care and examines the effect of a QI process on CCM involvement. We also aimed to characterize practice patterns and the impact of CCM consultation by assessing characteristics associated with CCM involvement and the impact of CCM involvement.

## Materials and methods

Institutional review board

This study (STUDY00009382) was approved on March 1, 2022, by the Institutional Review Board of the University of Washington, and a waiver of consent was in place for a retrospective analysis of data gathered between 1/1/2014 and 1/31/2021.

Study design and patient selection

We assessed the involvement of CCM in primarily Amharic/Cambodian/Khmer/Somali/Spanish/Vietnamese-speaking adult patients at a 413-bed, level-I trauma and designated Comprehensive Stroke Center with a 30-bed dedicated neurocritical care service. Patients were included and deemed “CCM eligible” if they or their families were identified as members of the respective community. We launched a quality improvement project in January 2019 to raise awareness and encourage healthcare providers to consider CCM referrals. The period of 1/1/2014 to 1/31/2018 was considered the pre-CCM project implementation period, and that between 1/1/2019 to 1/31/2021 was considered the post-CCM project implementation period. At our institution, CCM involvement can occur via 1) a request made by the healthcare team or 2) self-referral from the CCM team member after identifying the patient's preferred language in the electronic medical records (EMR). For this study, we retrospectively identified all potentially eligible patients by electronic chart review and compared clinical characteristics and outcomes between eligible patients who received CCM consultation and those who did not. We also compared CCM referral patterns before and after implementing our QI project.

Outcomes of interest

The primary outcomes were 1) rates of CCM involvement before and after the implementation of the QI project, 2) Differences and outcomes between CCM-eligible patients who received CCM consultation versus those who didn’t, 3) Qualitative analysis of the CCM role, concerns shared by patients/families and support offered by the CCM team, which was gathered from CCM progress notes entered in the EMR and direct feedback from the CCM team.

The structure and function of the CCM team

The CCM team comprises hospital employees representing five communities of NELP patients and families: Amharic, Cambodian/Khmer, Somali, Spanish, and Vietnamese. Each representative is proficient in the language, has been born in the respective country, and shares the same cultural background. All CCMs receive advanced training in medical interpretation, cultural mediation, and case management, understand the role of the community advisor as a resource person, understand the resources and roles of the health and social institutions in the local area, interact with health care professionals, and reviewing cases with medical staff [10].

The CCM team is typically available for consultation with medical teams between Monday and Friday during the daytime. While not routinely available on the weekend, exceptions are made for complex cases, and the critical care team may contact the CCM team. As part of the internal CCM self-referral process, the CCM team reviews the list of all hospitalized patients daily to identify patients/families/surrogate decision-makers they may be able to support.

Following consultation or self-referral, the CCM approaches the patient/families/surrogate decision-maker, provides education regarding the role of the CCM team, and offers involvement of the CCM team. CCMs are responsible for documenting patient encounters in the EMR. Once a referral is received, and the family has agreed to CCM involvement, the CCM will meet with the care team and delineate an individualized plan regarding communication and multidisciplinary family meetings. During these meetings, families receive educational material related to critical illnesses or death by neurological criteria (DNC).

Communication with the care team is essential to CCM workflow. CCM add themselves to the patient's care team list in the EMR and document family meetings and phone calls with patients/families. This establishes continuity of care and informs other healthcare team members about their involvement. The workflow of the CCM encompasses the following standards of practice: Navigation, Case Management, Mediation/Advocacy, Education, and Interpretation. CCM seeks out resources in the patient’s community that provide cultural, religious, and social support and often partners with religious leaders. In addition to direct patient contact, CCM interacts with outside hospitals, schools, and social organizations and organizes health fairs in their communities. Thus, the CCMs offer a wrap-around service by connecting patients and families to internal and external resources.

Outline of the QI project

**Figure 1:**
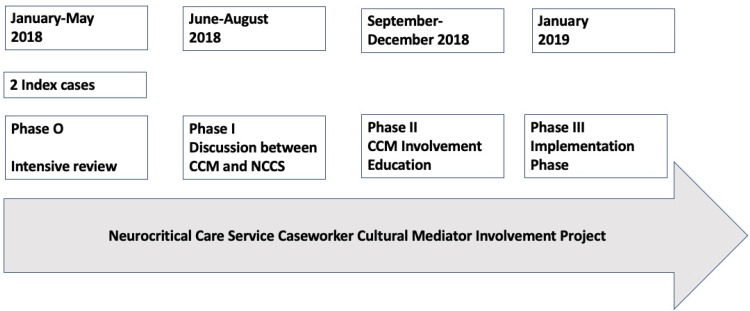
Phases and Timeline for the Neurocritical Care Service Caseworker Cultural Mediator (CCM) Involvement Project CCM: Caseworker Cultural Mediator; NCCS: Neurocritical Care Service

Figure 1 describes the three phases of the project.

Phase 0 consisted of a review of two index cases involving two patients belonging to NELP and CCM-eligible communities who were declared DNC, resulting in contentious discussions with the care team after the family had a difficult time coming to terms with the medical diagnosis. The CCM was involved late in the process after communication breakdowns had occurred and provided remediation support to healthcare providers and families. This was the first time our neurocritical care team recognized the importance of the CCM team’s expertise in supporting NELP patients and mediating conflict.

Phase I consisted of several rounds of discussions between the neurocritical care team and members of the CCM team to delineate synergistic interactions between the CCM and the healthcare team. Initially, the author AVL met with the CCM team to obtain input regarding the perceptions of CCM-eligible patients towards end-of-life care and DNC. Several themes emerged, such as insufficient time between admission and neurological prognostication, insufficient time for the family to process the loved one’s condition, and perceived lack of accommodation by the neurocritical care service of the requests by families/surrogate decision-makers towards the continuation of care/somatic support. At the end of these discussions, a plan was made to invite CCM team members to a monthly multidisciplinary neurocritical care service meeting to enhance regular dialogue between the CCM and neurocritical care teams.

Phase II consisted of educational sessions with the CCM team. The neurocritical care service put together a proposal to incorporate the benefits of the CCM team in routine neurocritical care workflow. It was decided that prompt consultation with the CCM team would occur within 24 hours of the patient’s admission to the neurocritical care service.

We created flyers that included the project proposal, contact information for the project leads from neurocritical care service and CCM teams, authors AVL and LAM, respectively, and held several educational sessions.

Simultaneously at this time, the hospital’s DNC policy underwent a revision. Due to the two index cases, it was decided to include a multidisciplinary huddle before the initiation of DNC determination with the involvement of the CCM team before approaching families/surrogate decision-makers regarding DNC, as outlined in the World Brain Death Project [9].

Phase III

We collected data on the involvement of the CCM team, time from admission to the participation of the CCM team, as well as progress noted by CCM, neurocritical care service, nurses, and social workers.

Data Collection

Patient information and specific data were collected from the EMR and refined via a departmental database, Perioperative & Pain Initiatives in Quality Safety Outcome (PPIQSO). The data points collected were as follows:

1) Patient characteristics: age, sex, admitting diagnosis, race, ethnicity, preferred language, insurance carrier, admitting Glasgow Coma Scale score (GCS), the maximum (SOFA) scores, need and duration of mechanical ventilation, intensive care unit (ICU) and hospital length of stay (LOS), all-cause in-hospital mortality, transition to comfort measures only (CMO) and DNC.

2) Caseworker cultural mediator (CCM) services: We screened the EMR for the consultation notes by members of the CCM team. We noted the date and time the first note was written and calculated the time since admission to the consultation note (in days).

3) Authors AVL and AB met with members of the CCM team over a Zoom^TM^ (San Jose, CA, USA) conference call to obtain insight from CCM team members about a priori selected topics: 1) common concerns voiced by the CCM-eligible patients/families/surrogate decision makers, 2) common requests made by CCM-eligible patients/families as relayed by CCM team members, 3) and discussed proposed solutions.

4) Author AB extracted clinical notes for all CCM and neurocritical care service encounters for the corresponding days from the study participants from the electronic medical record and saved them as Microsoft Word documents.

5) We examined the notes for follow-up meetings with patients/families or a lack thereof.

Statistical Analysis

The descriptive analysis described the study cohort characteristics. Categorical variables were expressed as counts and percentages. The Shapiro-Wilk test was performed to examine the normality of distribution of continuous variables such as age, intensive care unit, and hospital length of stay expressed as mean+SD or median [interquartile range, IQR]. Differences between patient characteristics in the pre-and post-implementation periods were performed using a two-tailed t-test and Chi-square test and calculated odds ratios and 95% confidence interval (CI). We also compared the patients in whom CCM self-referral led to families declining their request to be involved in the patient's care. We used the Chi-square test to understand the temporal trends in CCM involvement between 2014 and 2021. In multivariate models, we examined characteristics of patients who received CCM among CCM-eligible cases, associations between CCM involvement and transition to CMO, and associations between CCM involvement and ICU LOS after adjusting for age, admission GCS, mechanical ventilation, and maximum SOFA scores. We reported the results as adjusted odds ratios (aOR) and corresponding 95% CIs. Multivariate models were tested for Hosmer-Lemeshow goodness-of-fit test. A Bonferroni-corrected p-value of < 0.05 was considered to be statistically significant. Qualitative analysis pertained to the common themes identified after discussion with CCM team members and after a review of clinical notes. We codified major and minor themes and presented them as counts and percentages. STATA version 15 (StataCorp LLC, College Station, TX, USA)/RStudio 1.554 (R Foundation for Statistical Computing, Vienna, Austria) was used for statistical analysis [11,12].

## Results

Pre-implementation and post-implementation comparison

Table 1highlights differences in the characteristics of the patients before (n=579) and after implementation (n=369) of the neurocritical care service CCM involvement project. After both samples were matched for age, gender, admitting diagnoses, mechanical ventilation status, admission GCS, maximum SOFA scores, ICU/hospital LOS, and DNC, we observed two key differences. First, CCM involvement was higher in the post-implementation sample (22% vs. 6.91%, OR 3.78, 95% CI [2.53; 5.71], p < 0.001). Second, there were higher rates of CMO in the post-implementation sample (9.21% vs. 5.35%), OR 1.79 95% CI [1.08; 2.99], p=0.031).

**Table 1:** Differences in Patient Sample Characteristics Before and After Implementation of the Caseworker Cultural Mediator Involvement Quality Improvement Project ICU: intensive care unit; LOS: length of stay; SOFA: Sequential Organ Failure Assessment; CMO: comfort measures only

	Pre-implementation period	Post-implementation period	Odds Ratio for CCM involvement (95% Confidence interval)	Bonferroni-corrected p-value
	N=579	N=369		
Caseworker Cultural Mediator Involvement	40 (6.91%)	81 (22.0%)	3.78 [2.53;5.71]	<0.001
Age in years	54.3 (17.7)	54.4 (18.1)	1.00 [0.99;1.01]	0.982
Sex				0.847
Female	223 (38.5%)	139 (37.7%)	Reference group	Reference group
Male	356 (61.5%)	230 (62.3%)	1.04 [0.79;1.36]	0.795
Insurance:				0.796
Commercial	88 (15.2%)	53 (14.4%)	Reference group	Reference group
Non-commercial	491 (84.8%)	316 (85.6%)	1.07 [0.74;1.55]	0.729
Admitting diagnosis				
Acute ischemic stroke	172 (29.7%)	101 (27.4%)	Reference group	Reference group
Others	53 (9.15%)	13 (3.52%)	0.42 [0.21;0.79]	0.007
Postoperative care	82 (14.2%)	60 (16.3%)	1.25 [0.82;1.88]	0.300
Spontaneous subarachnoid hemorrhage	76 (13.1%)	44 (11.9%)	0.99 [0.63;1.54]	0.954
Spontaneous intracerebral hemorrhage	67 (11.6%)	63 (17.1%)	1.60 [1.05;2.45]	0.030
Status epilepticus	14 (2.42%)	13 (3.52%)	1.58 [0.70;3.54]	0.266
Traumatic brain injury	103 (17.8%)	64 (17.3%)	1.06 [0.71;1.57]	0.780
Traumatic spinal cord injury	12 (2.07%)	11 (2.98%)	1.56 [0.65;3.72]	0.315
ICU length of stay (days)	6.09 (7.54)	5.94 (13.0)	1.00 [0.99;1.01]	0.820
Hospital length of stay (days)	15.8 (29.1)	17.8 (35.2)	1.00 [1.00;1.01]	0.349
Admission Glasgow Coma Scale score	11.1 (4.52)	10.5 (4.79)	0.97 [0.94;1.00]	0.033
Maximum SOFA score	4.51 (3.28)	4.60 (3.43)	1.01 [0.96;1.06]	0.780
Mechanical Ventilation status	238 (41.1%)	171 (46.3%)	1.24 [0.95;1.61]	0.129
All-cause in-hospital mortality	70 (12.1%)	53 (14.4%)	1.22 [0.83;1.79]	0.359
Transition to CMO	31 (5.35%)	34 (9.21%)	1.79 [1.08;2.99]	0.031
Death by Neurologic Criteria	26 (4.49%)	13 (3.52%)	0.78 [0.38;1.52]	0.573

Temporal trends in caseworker cultural mediator involvement in neurocritical care

**Figure 2:**
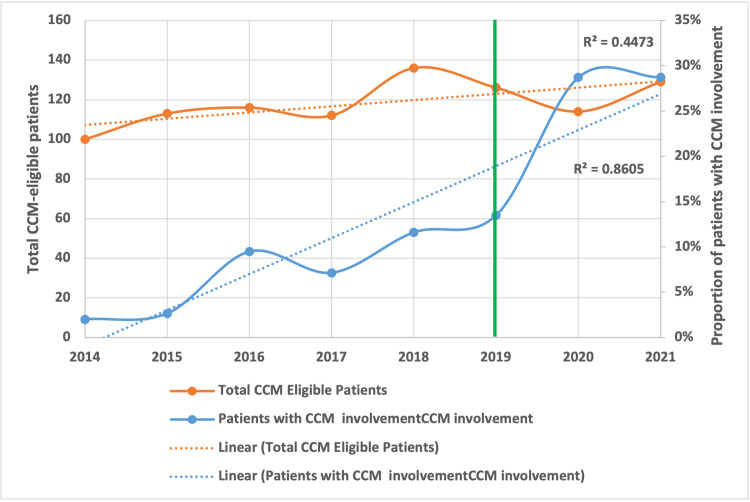
Trends in Caseworker Cultural Mediator Involvement in the Care of Patients Requiring Neurological/Neurosurgical Critical Care The green line indicates the go-live for the CCM involvement project

Figure 2 represents a run chart of the trends in CCM involvement over the study period.

There was a statistically significant increase in CCM involvement from 2014-2021, a point estimate of 0.33, OR 1.39, the area under the curve, 0.69, p < 0.001. The median time from admission to CCM involvement was 2.2 days [IQR 8.95]. The time to CCM involvement in the pre-implementation (2.3 days [IQR 11.3]) and post-implementation period (2.3 days, [IQR 8.3]) was similar (p=0.25). Numerically, the time from admission to CCM involvement was shorter in patients who transitioned to CMO compared to those who didn’t (1.45 days [IQR 3.25] vs. 2.4 days [IQR 10.4]); however, this was not statistically significant (p=0.58).

Differences between CCM-eligible patients with versus without CCM involvement

Table 2 summarizes differences between patients with CCM involvement and those without CCM involvement among all CCM-eligible patients. Patients with CCM involvement were younger (49 [IQR 38,63] years vs. 56 [IQR 42,68] years), p=0.002), had greater illness severity (admission GCS 8.5 [IQR 3,14] vs. 14 [IQR 7,15], p < 0.001), higher SOFA scores (5 [IQR 2,8] vs. 4 [IQR 2,6], p=0.007), and more frequently required mechanical ventilation (67% vs. 40%, OR 3.07, 95% CI 2.06, 4.64]). Numerically, DNC patients were more likely to receive CCM involvement, but this was not statistically significant (5.79% vs. 3.87%, OR 1.55 [IQR 0.61,3.42], p=0.33).

**Table 2:** The Differences in the Caseworker Cultural Mediator-Eligible Patients Who Received Caseworker Cultural Mediator Involvement and Who Did Not SOFA: sequential organ failure assessment; LOS: length of stay; GCS: Glasgow Coma Scale

	No CCM involvement	CCM involvement	The odds ratio for CCM involvement (95% confidence interval)	Bonferroni corrected p-value
	N=827	N=121		
Age (years) mean (SD)	55.0 (17.9)	49.7 (17.2)	0.98 [0.97;0.99]	0.002
Gender:				
Female	321 (38.8%)	41 (33.9%)	Reference group	Reference group
Male	506 (61.2%)	80 (66.1%)	1.24 [0.83;1.86]	0.299
Insurance carrier:				1.000
Commercial	123 (14.9%)	18 (14.9%)	Reference group	Reference group
Non-commercial	704 (85.1%)	103 (85.1%)	0.99 [0.59;1.75]	0.981
Admission GCS mean (SD)	11.2 (4.53)	8.81 (4.85)	0.90 [0.87;0.94]	<0.001
Maximum SOFA score mean (SD)	4.34 (3.22)	5.49 (3.75)	1.10 [1.03;1.17]	0.003
Mechanical ventilation status	328 (39.7%)	81 (66.9%)	3.07 [2.06;4.64]	<0.001
NICU LOS (days) mean (SD)	5.48 (8.99)	9.86 (14.8)	1.03 [1.01;1.05]	0.001
Hospital LOS (days) mean (SD)	13.5 (23.1)	37.2 (61.0)	1.02 [1.01;1.03]	<0.001
All-cause in-hospital mortality	99 (12.0%)	24 (19.8%)	1.83 [1.09;2.95]	0.022
Transition to comfort measures only	51 (6.17%)	14 (11.6%)	2.00 [1.03;3.66]	0.040
Death by Neurologic criteria	32 (3.87%)	7 (5.79%)	1.55 [0.61;3.42]	0.332

**Figure 3:**
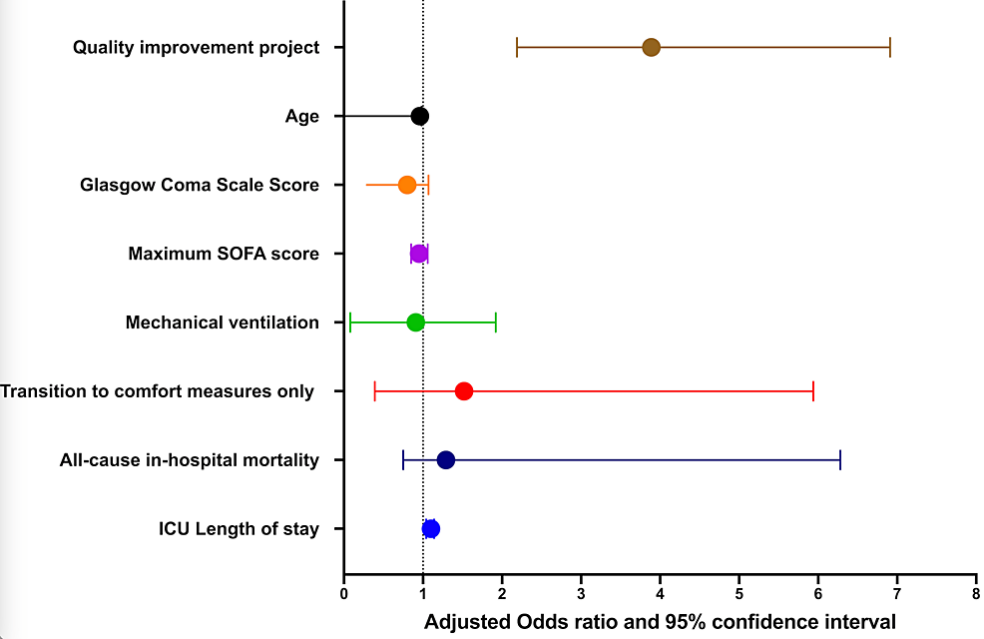
Multivariate analysis Examining Reasons for Caseworker Cultural Mediator Involvement in Neurocritical Care ICU: intensive care unit: SOFA: Sequential Organ Failure Assessment

On multivariate analysis after adjusting for age, admission GCS, Maximum SOFA score, ICU LOS, all-cause in-hospital mortality, and transition to CMO, implementation of the CCM QI project was independently associated with CCM involvement (aOR 4.22, 95% CI [2.32;7.66], p < 0.001, Figure 3, Hosmer-Lemeshow goodness-of-fit test, p = 0.1178).

Associations between CCM involvement, CMO transitions, and ICU LOS

Patients with CCM involvement had longer intensive care unit stay (5 [IQR 2,13] days vs. 3 [IQR 2,5] days) and hospital length of stay (18 [IQR 7,40] days vs. 7 [IQR 3,16] days), with higher all-cause mortality (20% vs. 12%, RR 1.83, 95% CI 1.09, 2.95), and with a higher rate of transition to CMO (11.6% vs. 6.2%, OR 2.00, 95% CI 1.03;3.66, p=0.045).

However, in a multivariate model adjusting for age, mechanical ventilation, admission GCS, and maximum SOFA scores, CCM involvement was not associated with an increased likelihood of transition to CMO (aOR 1.53, 95 CI [0.64; 3.71], p = 0.34, Hosmer-Lemeshow goodness-of-fit test, p = 0.5432).

Also, multivariate analysis adjusting for age, mechanical ventilation, admission GCS, and maximum SOFA scores suggested that CCM involvement was less likely to prolong ICU LOS (aOR 0.62, 95% CI [0.49;0.79], p=0.0001, Hosmer-Lemeshow goodness-of-fit test, p = 0.55).

Qualitative analysis of CCM involvement

**Figure 4:**
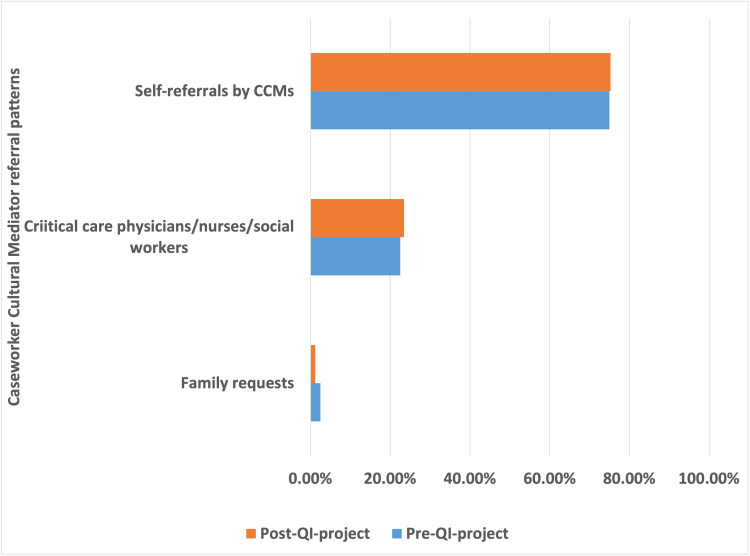
Caseworker Cultural Mediator Involvement Referral Patterns in Patients Requiring Neurological/Neurosurgical Critical Care

As Figure 4 shows, there was a proactive approach by the CCM team to educate families about the role of CCM by self-referral (n=91, 75.2%). Only a minority of consultations originated from critical care physicians and nurses (n=28, 23.1%) and families (n=2, 1.65%). The proportion of self-referrals (pre: 75% vs. post: 75.3%), critical care physicians/nurses requests (pre: 22.5% vs. post: 23.5%), and family requests (pre: 2.5% vs. post: 1.2%) remained consistent in the pre-and post-QI project implementation periods. Overall, 38% (n=43) of CCM team referrals did not result in any follow-up requests by the family. The patients in whom family followed up with CCM team member were related to their admission GCS (OR 0.92, 95% CI [0.85,0.99], p=0.03), being mechanically ventilated (OR 2.66, 95% CI [1.22,5.92], p=0.02), and with all-cause in-hospital mortality (OR 2.76, 95% CI [1.05,8.29], p=0.04).

CCMs reported providing cultural/emotional support (n=96, 79%), end-of-life counseling (n=16, 13%), provided conflict mediation (n=15, 12.4%), and facilitated goals of care meetings (n=4, 3.3%). Specifically, CCMs provided cultural and linguistic care mediation, facilitation of communication within the neurocritical care team and families, provision of resources for rehabilitation and long-term condition management, location of housing and long-term care facilities, providing letters of support for out-of-the-country family members wishing to travel to the United States, and advocating for patient and family wishes. CCMs also assisted with locating legal next of kin and in the determination of childcare for minors.

**Figure 5:**
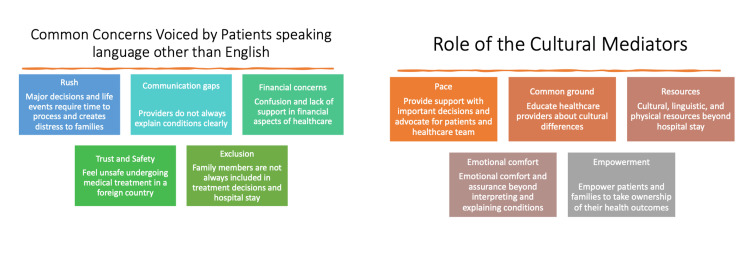
Common Concerns Voiced by Caseworker Cultural Mediator-Eligible Patients/Family and Solutions Proposed by the Caseworker Cultural Mediator Consultation

Figure 5 highlights common concerns voiced by NELP patients/families and support provided by the CCM. The main areas of problems for which CCM provided support were as follows: families expressed feeling rushed in making life-or-death decisions, communication gaps, financial concerns due to different healthcare systems in the US compared to their native country, patients/families expressed feeling unsafe in a foreign country and feel excluded in treatment decisions and hospital stay.

We present themes and quotes from the CCM progress notes in Table 3, which highlight the common concerns and solutions provided by CCMs in the following areas: language barriers causing anxiety, inadequate housing, emotional distress due to patient's condition, demographic and personal specifics of the patient, cultural values, legal, financial, and next-of-kin mediation, medical procedures, resources for patient families, the impact of COVID-19 visitation rules, and services to assist with external concerns.

**Table 3:** Quotes from Caseworker Cultural Mediator Notes

Concerns addressed	Quote from Caseworker Cultural Mediator (CCM) Notes	Common solutions offered by CCMs
Language barrier causing care anxiety	"…[family member] understood the word "surgery" among other words. She told the voice [on the phone] that she understands very little English", "… [family member] reported that her daughter called her and informed her that the surgeon had found "something" in her liver," "[patient's father] called asking questions about some an earlier call he received regarding some PPW. [patient's father] is very confused at this point," "[patient's brother] understands and speaks English but has problems, sometimes, with the medical lingo," "Once [patient] understood what Speech and Respiratory staff was trying to do... patient collaborated with staff."	Linguistic and emotional mediation; explanation of conditions from the care team
Inadequate housing	"…[patient's mother] share[d] with me the issues that she has her current housing and how her daughter can’t stay with her since she has a daycare business in her home", "[patient's family member asked] if her request for housing was still in place and if we knew if they get any help, they live ... far away from Seattle", "[patient] relayed the story of being infected by black mold at a house he was living."	Collaboration with social workers to acquire housing or long-term care facilities
Emotional distress due to the patient's condition	"… [patient's father] stated, “I don’t know what has happened, but I just want my son to come home healthy as he has always been” [patient's mother] has felt a lot of pressure and judgment from her family for considering the possibility that [patient] might not recover," "[patient's mother] shared that she felt grateful to have a space where she could be heard and validated in these feelings," "[patient] hopes that she will get better, stronger so she can go home," "[patient] is the main caretaker of his family and was worried how his recovery will look like," "[patient's wife] has noticed changes in her husband—gets angry quickly and impatient.[She says:] “He was never like that.”... [patient's wife] reports [that] before surgery he was very independent and self-sufficient, so it is hard for him to agree to help", "[patient's daughter] expressed her fear of failing to take appropriate care of her mother if she is discharged to go home," "[patient's friend] talked about the spirits coming back to haunt her and the medical team if something went wrong," "Patient's wife shared some family pictures with me. I printed them out to create an album, which I brought to [patient] this afternoon ... As I showed him the pictures of his children and his wife, [patient] started to cry and leaned against me looking for support ... I called his wife ... [patient's wife] spoke to him, and he said to her the word AMOR, which means LOVE in Spanish."	Emotional and spiritual support; reassurance that the care team will do everything they can to help the patient
Demographic and personal specifics of the patient	"...this patient has two words, last name. In this culture, an individual's last name is formed by: two words; the first word of the father's last name and it continues with the first word of the mother's last name", "the wife indicated to us that her husband prefers to go by the nickname," "When examining sensation on the patient's left side extremities and asking where he could feel touch, I noticed that he would identify his arm as his 'mano,' or hand, in Spanish," "[patient] said he would prefer a male caregiver since [for] his bathroom needs [he] can’t have [a] female caregiver," "[patient and her daughter] have such a relationship that she knows she will know when her mother will be ready to go."	Specifying patient's preferred name on the chart; providing specific spiritual care; ensuring effective interpretation in cultural context
Cultural values	"Tonight is a holiday Hispanics celebrate. It is called Nochebuena. This is a day our families gather, so [patient's mother] is especially grateful that the nurse opened the Zoom call for her." "The family has communicated a clear and consistent preference not to remove mechanical ventilation because their faith does not recognize death by neurologic criteria. They seek more time for prayer."	Explanation of cultural peculiarities to the care team; mediation of care; advocacy for the patient and their family
Legal, financial, and next-of-kin mediation	"We talked about the LNK role and how despite our cultures outside of the United States. The LNK is the spouse if they are legally married, and after the spouse, parents will be LN," [patient's father] expressed concerns about care affordability for his daughter."	Assistance with locating legal next of kin; provision of letters of support for Embassies in foreign countries
Medical procedures	"The family reported being surprised about the autopsy need because they were told he suffered a brain aneurysm and was confirmed brain dead," "they reported the organ procurement staff came and asked about organ donation. At this time, they became mistrustful of the intent of the autopsy", "as a mother, she wanted to watch the autopsy to assure none of his organs were removed. She stated she felt that even though she had refused organ donation, she believed his organs would be taken during the autopsy", "I was able to clarify patient's brain death prognosis, as well as the nature of the neurological tests and ultrasound, performed today," "[patient's husband inquired] about the reason why the tumor was not removed completely," "we will discuss with providers the possible rearrangement of patient's medicines so he can be less agitated at night and sleep better," "[patient's wife] reported that she saw her husband removing his helmet yesterday during Zoom visit and scratch the incision in his head."	Explanation of care plan; involvement of the patient's family in the decision-making process; informing care team about important comments made by family members
Resources for patient families	"The children haven’t been at school since their mother’s accident… [family member] was worried [about] the kids' wellbeing and asked [ed] me how to start the process of temporary guardian[ship] until the mother recovers", "I asked about his [patient's relative] own health issues if he had any, I found he has diabetes, and he supposed to take at least three different medicines, but he is just taking only one and not as the medicine was prescribed," "[patient's caregiver] reported being a little bit confused because he was told what equipment his brother would need and then the list change[d]."	Ensuring proper medication adherence; updating family members on patient's health status; provision of accommodations for families in the hospital
Impact of COVID-19 visitation rules	"[family members] were upset about this new Harborview visitation rule and said that they understand the protection and safety from the family, patient, and the staff of the hospital from coronavirus but still unacceptable to be told that they can't see briefly their love[d] one... it's important for their love[d] one for healing to hear their voice, feel their touch and their presence", "[patient's sister] reported that she has noticed how patient's behavior improves when one of his siblings is at the bedside."	Mediation between the care team and families; facilitation of virtual family meetings
Services to assist with external concerns	"[patient's mother] is requesting a letter explaining the duration of his hospital stay at Harborview," "[patient] lost her prescription glasses while moving and asked me to contact eye clinic and mailed copy of the prescription," "[patient's] siblings want to come to see him, but he told them that it is better not to come because he worries that traveling can be a dangerous for them because of immigration," "Patient wishes to get one of her children here to visit and take care of the patient while she is in this medical condition," "If it’s possible, he [patient's son] would want to take his father back to Vietnam where he can take care of his father," "I explained that the more relatives we want to bring the more difficult to secure visas due to the immigration policies," "the [family asked] questions about funeral services, corpse preparation to take her to Puerto Rico."	Provision of physical resources for patients beyond hospital stay; assistance in scheduling appointments; repatriation of the patient

## Discussion

A single-center quality improvement (QI) project was undertaken to explore and increase the involvement of Caseworker Cultural Mediator (CCM) to provide culturally sensitive care for Non-English Language Preference (NELP) patients requiring neurocritical care, to bridge communication gaps and enhance the bond between NELP-patients, their families, and the medical team. Our main finding was that implementing the QI project was independently associated with increasing CCM involvement. Other results include 1) CCM involvement preferentially occurred in the NELP patients with higher illness severity, 2) CCMs were more likely to be involved with NELP patients undergoing a transition to CMO, and those with longer ICU LOS, however, when adjusted for illness severity, these associations became non-significant, 3) four out of ten attempts made by CCMs to reach out to the family to provide support were declined by the family, 4) CCMs reported providing cultural/emotional support, end-of-life counseling, conflict mediation, and facilitated goals of care meetings.

A multiphase QI project successfully increased the involvement of CCMs in the care of NELP patients. The individualized awareness of and attention to the needs of patients provided with the help of cultural mediators is integral to understanding the unique needs of our increasingly diverse populace. Patient differences in race/ethnicity can affect patient evaluations of care in many ways, potentially resulting in healthcare disparities. In a study comparing patient-centered care from the viewpoint of cancer patients of different ethnicities and races, unmet needs for time to make decisions and provider interaction between visits emerged among Blacks and Caucasian patients. At the same time, value for optimism was observed among Hispanic patients [13]. By creating awareness and bridging gaps in knowledge about the CCM team’s role on the part of the healthcare team, we attempted to address the lack of guidelines and protocols regarding CCM consultation triggers and successfully reduced the potential for missed opportunities. However, more work needs to be done in this area. Given the low rate of CCM involvement and a sizeable NELP population that can potentially benefit, we suggest solutions to increase consistency and sustainability in the participation of the CCM team. These solutions are aimed at overcoming communication barriers and bridging cultural gaps. This could be achieved by monthly educational opportunities for neurocritical care healthcare providers, including mandatory virtual/on-demand training, setting up institutional guidelines, and a consultancy request through the EMR, which was not present at the time of the study. Finally, adequate hospital resources should be reserved for the growth and sustenance of the CCM team and for exploring ways to expand services to other NELP communities beyond those currently supported.

Our study finds that CCM involvement was utilized for the sickest NELP patients and end-of-life support, including transitions to CMO and patients with longer ICU LOS. Since prognostication after neurological and neurosurgical emergencies often involves complex and time-sensitive conversations with the patients/families/surrogate decision makers, the CCM team may be seen as a “last resort” or after a communication breakdown with patient/patient families instead of employing a proactive approach to address and overcome language, cultural and religious barriers. This argument is highlighted by the two index cases where the CCM team had to resort to remediation regarding complex medical decision-making. Our study indicated that CCM involvement was not associated with an increased likelihood of CMO transitions and non-CMO in-hospital mortality when adjusted for illness severity. The project also highlights that within the construct of CMO discussions, without influencing the outcome (CMO or ICU LOS), the involvement of the CCM team may provide opportunities to provide culturally sensitive care to NCC patients in a time-sensitive manner.

The majority of CCM involvements included a proactive approach by the CCM team to identify patients based on their preferred language listed in EMR. The CCM team members introduced themselves to the patients/families, educated them on the support that could be provided, and left their contact information. In a minority of cases, we found that the social worker/bedside nurse/case manager/primary critical care team members reached out to CCM. The few times the critical care team reached out to CCM were in the context of end-of-life support and DNC. One case of DNC highlighted challenges in the accommodation of family requests after the diagnosis of the DNC was completed by the neurocritical care service. It underlined the importance of a proactive multidisciplinary approach to culturally sensitive care, as highlighted and recommended in the World Brain Death Project [9].

Medical care for NELP patients and families is stressful in various aspects, only one of which is the barrier of language [14]. CCMs likely contribute to their sense of control and support and provide solutions to real problems such as finance and housing. We were surprised to find that 4 out of 10 self-referrals by the CCMs resulted in a lack of a request by the NELP families to seek the support of CCM. The exact reasons for this are unknown, but our study suggests that in patients with high illness severity, families may reach out to CCMs for support when faced with challenges related to end-of-life/goals of care conversations. Future studies may examine why NELP families may not seek CCM support in neurocritical care.

Limitations

Several limitations are apparent, mainly because this was a single-center, nonrandomized quality improvement project with before/after comparison. Most importantly, this project did not examine patient/family-centered outcomes; thus, it was impossible to compare perceptions of the family/surrogate decision-makers towards culturally sensitive care provided with and without the involvement of CCMs. This low rate may be multifactorial such as lack of awareness and participation of CCMs only if there is a conflict between the neurocritical care provider and the families/surrogate decision makers. Another limitation of the current system at HMC for CCMs to know about potential patients/family needing their services (proactive approach) is that the CCMs only know of patients needing their services based on the patient's language as listed in the EMR, which may not always be recorded accurately. It does not factor in their families’ preferred language. Unless CCMs get a referral from a critical care team member, the CCM team may be unable to identify these families prospectively. Hence, the results from our study may not be generalizable to adult NCC patients outside our hospital but provide a benchmark for other hospitals and neurocritical care units/services to compare against.

## Conclusions

A local QI project rollout increased the involvement of CCMs in neurocritical care. This project attempts to create awareness among neurocritical care healthcare providers on ways to support NELP patients and families of different cultural backgrounds. The study results are encouraging, and there is potential to spread this awareness to other NELP patients/families and contexts other than critical care environments. The lessons learned from this study informed the neurocritical care and CCM team members that 1) NELP patients/families need support in many areas, support that typically is not provided by a medical interpreter, 2) there may be patients/families that may decline to follow up with a CCM team member, 3) high illness severity, end-of-life and goals of care conversations may be seen as triggers for CCM support, and 4) If CCM resources are limited then we may focus attention on these areas of need to maintain the sustainability of CCM team to provide culturally sensitive care. The perceptions of NELP patients/families regarding routine involvement of CCM in neurocritical care and its impact deserve further examination.
